# Bilious Vomiting in the Newborn: A Three-Year Experience in a Tertiary Medical and Surgical Centre

**DOI:** 10.1155/2020/8824556

**Published:** 2020-10-13

**Authors:** Rebecca A Lee, Theodore Dassios, Ravindra Bhat, Anne Greenough

**Affiliations:** ^1^Neonatal Intensive Care Centre, King's College Hospital NHS Foundation Trust, London SE5 9RS, UK; ^2^Department of Women and Children's Health, School of Life Course Sciences, Faculty of Life Science and Medicine, King's College London, London SE5 9RS, UK; ^3^Asthma UK Centre for Allergic Mechanisms in Asthma, King's College London, London SE1 9RT, UK; ^4^NIHR Biomedical Research Centre based at Guy's, St Thomas' NHS Foundation Trust, King's College London, London SE1 9RT, UK

## Abstract

**Background:**

Bilious vomiting in the newborn is common and requires urgent attention to exclude malrotation. The proportion of neonates with surgical abnormalities, however, is small, and there are other causes. *Study Objectives*. We reviewed our experience of infants with bilious vomiting to demonstrate the importance of input from the tertiary surgical and medical team to arrive at the correct diagnosis.

**Design:**

Admissions with bilious vomiting/aspirates of term born infants over a three-year period to a tertiary medical and surgical unit were reviewed.

**Results:**

During the study period, 48 infants were admitted with bilious vomiting. Forty-five infants had upper gastrointestinal (UGI) contrast studies, and only six had an abnormal study: four had malrotation and two had Hirschsprung's disease. Of the infants with a normal UGI study, no cause was identified in 20 cases, 13 infants were treated for sepsis, one had a meconium plug, one an ovarian cyst, and two infants were polycythaemic. One infant was diagnosed with bilateral polymicrogyria (PMG) on brain MRI and another was found to have hypochondroplasia FGFR3 skeletal dysplasia.

**Conclusion:**

Neonates with bilious vomiting may have a variety of underlying diagnoses and need to be referred to a tertiary surgical and medical centre to ensure appropriate diagnosis is made.

## 1. Introduction

Bilious vomiting or bilious aspirates in the newborn period requires urgent attention, as it may be the result of malrotation. Sadly, infants have died as a consequence of failure to promptly refer for appropriate investigation, as there is a predisposition inherent in malrotation to midgut volvulus threatening intestinal viability [1, 2]. The incidence of malrotation presenting in the neonatal period is estimated at 1 : 6000 live births [3]. An audit of bilious vomiting in term infants referred for paediatric surgical assessment demonstrated that only 11.7% had a surgical diagnosis, but no single test available in peripheral centres could have excluded a surgical diagnosis [4]. This audit demonstrated that there were a variety of nonsurgical diagnoses which were associated with bilious vomiting [4]. Similarly, in an audit of neonatal transport services and transfer for bilious vomiting, a surgical diagnosis was found in only 22% of cases [5] and various nonsurgical or no causes were found. We have reviewed infants referred to our tertiary surgical and medical centre to emphasize the wide range of diagnoses that can be associated with bilious vomiting, and hence, the importance of very thorough investigation of such infants should be performed by not only the tertiary surgical, but also the tertiary medical team.

## 2. Case Series

### 2.1. Method

All admissions of term born infants were reviewed over a three-year period (01 Jan 2017–31 Dec 2019) with bilious vomiting or bilious aspirates. Admission details, patient demographics, and final diagnoses following admission were collected via the Badger.net electronic resource. The results of radiological and haematological investigations undertaken were obtained from the electronic patient records.

## 3. Results

There were 48 infants admitted over the time period (26 were admitted from the labour ward, postnatal ward, or Accident and Emergency unit at King's College Hospital NHS Foundation Trust (KCH), and 22 were transferred from other neonatal units for assessment at KCH. Their median (or IQR) gestational age was 39 + 6 (39-40) weeks, their birth weight was 3432 (3240–3850) g and age at admission was 23.5 (19–44) hours. Their median duration of inpatient admission was four (range 2–7) days.

Three infants had no upper gastrointestinal (UGI) contrast study: one was diagnosed with anorectal malformation immediately following transfer, one had clinical features suggestive of Hirschsprung's which was subsequently confirmed on rectal biopsy, and one baby was diagnosed with sepsis immediately on transfer. An UGI contrast study was performed on the other 45 infants. Six infants had abnormal UGI contrast findings, four had a diagnosis of malrotation, and two were subsequently diagnosed with Hirschsprung's disease.

Of the infants with normal UGI studies, 20 infants (41.6%) had no further problems, 13 (27%) were treated for sepsis with a raised C-reactive protein (CRP), but with no positive blood cultures or cerebral spinal fluid (CSF) results, one had a meconium plug, one had an ovarian cyst, and two infants had polycythaemia which required treatment. Of the remaining two infants, one infant developed bilious vomiting on day two and seizures on day three. The male infant was born at 40 + 3 weeks of gestation, birth weight of 3850 g with no significant family history. Following his transfer to the tertiary NICU, he also had further seizure activity that clinically correlated with cerebral function monitor (CFM) changes. A brain MRI demonstrated bilateral polymicrogyria (PMG) affecting the frontoparietal and perisylvian regions. Karyotyping was normal. The other infant was born at 40 + 4 weeks of gestation, and the antenatal ultrasound examination at 36 weeks of gestation demonstrated long bones measuring below the third centile. A possible diagnosis of hypochondroplasia was suggested. She developed bilious vomiting at 24 hours and on day three episodes of apnoea, bradycardia, and desaturation which responded to intubation. She remained ventilated for 48 hours. There was no evidence of seizure activity on bedside CFM or electroencephalography (EEG). An MRI brain showed oversulcation in the medial temporal and occipital lobes in keeping with the diagnosis of hypochondroplasia. She was polycythaemic with a venous haematocrit of 71% on day three which responded to intravenous hydration. She was screened for sepsis, with no abnormal findings and completed a course of intravenous antibiotics. Hypochondroplasia FGFR3 skeletal dysplasia was subsequently confirmed from the results of antenatal genetic testing.

## 4. Discussion

We have demonstrated that bilious vomiting/aspirates in the newborn was associated with a variety of diagnoses. Others have reported unusual causes of bilious vomiting, but involving the gastrointestinal tract including intestinal duplications [6] and situs inversus totalis [7]. Other series have emphasized no underlying disorders, surgical abnormalities, or infection. Indeed, the bilious vomiting in 62% of infants in one series resolved with conservative management [8]. Our series emphasizes the importance of referring infants with bilious vomiting to a centre where both surgical and medical expertise is available as they had a wide variety of other conditions.

Forty-five of the 48 infants underwent an UGI contrast study. Such investigation is in line with current recommendations [9]. Indeed, in one series, barium contrast studies had a positive predictive value of 85.7% for surgical findings [10]. In a survey of practitioners in the west of Scotland, they generally did not consider an UGI contrast appropriate for a single bile vomit, but in a neonate with persistent bilious vomiting, paediatric surgical referral was considered the highest priority [11]. Clinical findings can determine whether affected patients have a time-critical condition, e.g., volvulus, where a delay in treatment is likely to compromise gut viability. Such clinical abnormalities include abdominal distension, abdominal tenderness, and abnormal radiograph findings [12]. Our series emphasize that infection must be considered and if suspected appropriately treated. Two of our infants had polycythaemia, and another had an ovarian cyst, but there are no reported mechanisms for the association. Our report emphasizes that infants with bilious vomiting, which persists despite a normal UGI, should have further investigation.

PMG is a malformation of the developing brain characterised by abnormal cortical lamination and an unusual folding pattern of the cerebral cortex such that all or part of the brain surface is taken up by an excessive number of small gyri [13]. It accounts for approximately 20% of all malformations of cortical development [14]. The diagnosis is made by MRI as computed tomography, and other imaging methods do not have sufficient resolution to identify the small folds that define PMG. It is usually an isolated finding occurring in the absence of other brain malformations, as in the case we describe. Bilateral perisylvian PMG, the most common PMG pattern, is associated with oromotor dysfunction and seizure disorder. There can also be difficulties with sucking and swallowing, facial diplegia, and limb spasticity. Neurological examination of our case did not reveal any abnormalities. The bilious vomiting may have related to swallowing abnormalities as it settled on nasojejunal feeding. Making the diagnosis was important as 75% of patients have mild-moderate intellectual disability, and parents need appropriate counselling. Furthermore, a systematic review of 48 papers demonstrated expressive and receptive language impairment, and oral structural and functional deficits are frequent [15].

Pathogenic variants in the fibroblast growth factor receptor 3 (FGFR3) gene underlie a broad spectrum of skeletal dysplasia including hypochondroplasia [16]. The mutations constitutively activate the FGFR3 receptor causing abnormal membranous ossification, suppressing chondrocyte growth and proliferation, and ultimately hindering bone elongation. Hypochondroplasia is characterised by disproportionate short stature, macrocephaly, brachydactyly, limited range of motion at the elbows, lumber lordosis, and bowed legs. In biallelic FGFR3, severe obstructive sleep apnoea and focal migrating seizures can occur [16]. In our case, the EEG was normal, and insertion of the endotracheal tube resulted in cessation of the apnoeas suggesting they were obstructive apnoeas. We are unaware that this condition has previously been associated with bilious vomiting and thus suggest this was related to her polcythaemia, as was seen in several of our other cases. Indeed, there have been a number of reports of infants with polycythaemia who had poor feeding or vomiting [17, 18].

## 5. Conclusions

Our results highlight that a surgical diagnosis is uncommon in neonates with bilious vomiting. More importantly, we demonstrate that, in those infants with a normal upper gastrointestinal contrast study, further investigation and enhanced medical management may be required by the tertiary medical team.

## Data Availability

The data used to support the findings of this study are available from the corresponding author upon request.
